# Corticotropin-Releasing Hormone Receptor 1 Gene Variants in Irritable Bowel Syndrome

**DOI:** 10.1371/journal.pone.0042450

**Published:** 2012-09-05

**Authors:** Naoko Sato, Naoki Suzuki, Ayaka Sasaki, Emiko Aizawa, Takeshi Obayashi, Motoyori Kanazawa, Tomoko Mizuno, Michiko Kano, Masashi Aoki, Shin Fukudo

**Affiliations:** 1 Department of Behavioral Medicine, Tohoku University Graduate School of Medicine, Sendai, Japan; 2 Department of Neurology, Tohoku University Graduate School of Medicine, Sendai, Japan; 3 Department of Applied Informatics for Human and Life Sciences, Tohoku University Graduate School of Information Science, Sendai, Japan; University of Tennessee, United States of America

## Abstract

**Background:**

Corticotropin-releasing hormone (CRH) acts mainly via the CRH receptor 1 (CRH-R1) and plays a crucial role in the stress-induced pathophysiology of irritable bowel syndrome (IBS). Several studies have demonstrated that variants of the *CRH-R1* gene carry a potential risk for depression, but evidence for an association between *CRH-R1* genotypes and IBS is lacking. We tested the hypothesis that genetic polymorphisms and haplotypes of *CRH-R1* moderate the IBS phenotype and negative emotion in IBS patients.

**Methods:**

A total of 103 patients with IBS and 142 healthy controls participated in the study. Three single-nucleotide polymorphisms of the *CRH-R1* gene (r*s7209436, rs242924, and rs110402*) were genotyped. Subjects' emotional states were evaluated using the Perceived-Stress Scale, the State-Trait Anxiety Inventory, and the Self-rating Depression Scale.

**Results:**

The TT genotype of *rs7209436* (*P* = 0.01) and *rs242924* (*P* = 0.02) was significantly more common in patients with IBS than in controls. Total sample analysis showed significant association between bowel pattern (normal, diarrhea, constipation, or mixed symptoms) and the T allele of *rs7209436* (*P* = 0.008), T allele of *rs242924* (*P* = 0.019), A allele of *rs110402* (*P* = 0.047), and TAT haplocopies (*P* = 0.048). Negative emotion was not associated with the examined *CRH-R1* SNPs.

**Conclusion:**

These findings suggest that genetic polymorphisms and the *CRH-R1* haplotypes moderate IBS and related bowel patterns. There was no clear association between *CRH-R1* genotypes and negative emotion accompanying IBS. Further studies on the CRH system are therefore warranted.

## Introduction

Impact of stress on human life is getting more recognized all over the world. A hallmark of the stress response is the activation of the autonomic nervous system and hypothalamo-pituitary-adrenal (HPA) axis [1]. The organism needs the normal stress hormone response to survive difficult situations, and inadequate or excessive adrenocortical and autonomic function is deleterious for health and survival [1]. The individual stress response via the corticotropin-releasing hormone (CRH) system is highly likely to affect the features of many stress-related disorders [2]. CRH is secreted from the paraventricular nucleus of the hypothalamus in response to stress [3]. CRH binds to CRH receptors that initiate the stress response leading to release of adrenocorticotropic hormone (ACTH) from the anterior pituitary lobe and simulation of serum cortisol secretion from the adrenal cortex [4]. The effect of CRH is mediated via CRH receptors in the cell membrane of effecter organs [5]. CRH receptors comprise seven-transmembrane G-protein coupled receptors [5]. Activation of CRH receptors stimulates adenylate cyclase activity increasing cyclic adenosine monophosphate (cAMP) levels in anterior pituitary corticotrophs resulting in ACTH release [6].

Irritable bowel syndrome (IBS) is a prototypic functional gastrointestinal (GI) disorder [7] generally accompanied by visceral hypersensitivity [8], increased gut reactivity [9], and altered central processing [10] in response to various stressors [11], [12]. Patients with IBS often have psychological abnormalities which are manifested mainly by increased levels of anxiety and depression [13]. The stress response via CRH release is highly likely to affect the features of IBS [4]. Exogenous administration of CRH mimics features of IBS in rodents [14] and humans [9]. Moreover, exogenous administration of CRH exaggerates central [9], [15] and gastrointestinal [9] responses in IBS patients, while peptidergic CRH antagonists reverse these phenomena [16], [17]. Taken together, these findings suggest that CRH plays a major role in the pathophysiology of IBS. Two major CRH receptors, CRH-R1 and CRH-R2, have been identified and have functional differences between them [2]. Stimulation of CRH-R1 causes anxiety, whereas that of CRH-R2 induces anxiolysis. CRH-R1 stimulation evokes colonic motility and mediates visceral nociception. In contrast, CRH-R2 stimulation inhibits gastric emptying and may reduce visceral perception [4]. Activation of CRH-R1 causes a proinflammatory response, whereas stimulation of CRH-R2 provokes anti-inflammatory changes [2]. Treatment with a specific CRH-R1 antagonist attenuates anxiety and increases colonic motility under stressful conditions after colorectal distention in rats [18]. Another specific CRH-R1 antagonist also reduced the increased brain activation in response to expected threats in IBS patients compared with a placebo [19]. These studies suggest that CRH signals via CRH-R1 are likely to be a key determinant of brain-gut function in response to stress in IBS patients.

The gene encoding CRH-R1 is located on chromosome 17q21.31 and contains 14 exons spanning 51 kb [20], [21]. Variation in the *CRH-R1* gene has been found to be a risk for depression following childhood maltreatment [22]–[25]. The variability of genes that encode the proteins which play a pivotal role in regulating the HPA axis influence the inter-individual clinical response to antidepressants [26], [27]. Previous study from our laboratory reported that less maternal care and maternal overprotection form risk for IBS-like symptoms in 7-year old children [28]. Earlier studies have reported moderation of the effects of maltreatment on depression and neuroticism by a three-allele haplotype of *CRH-R1* involving the single-nucleotide polymorphisms (SNPs) *rs7209436*, *rs110402*, and *rs242924* [22], [23], [29]. In these studies, the TAT haplotype protected against depression in individuals who had been severely maltreated. These findings led us to predict that the *CRH-R1* SNPs and the TAT haplotype might be associated with IBS and/or negative emotion in IBS patients.

In the present study, we investigated the association between variation in three *CRH-R1* SNPs and the presence of IBS or negative emotion in patients with IBS. We hypothesized that genetic polymorphisms and/or haplotypes of *CRH-R1* may moderate the effects of IBS symptoms as well as depression or anxiety in IBS patients. Our findings suggest that genetic polymorphisms and the *CRH-R1* haplotypes moderate IBS and related bowel patterns, although there was no clear association between *CRH-R1* genotypes and negative emotion accompanying IBS.

## Materials and Methods

### Subjects

In total, 103 patients (43 males and 60 females) with IBS who were diagnosed at the Department of Psychosomatic Medicine, Tohoku University Hospital, were enrolled in the study (mean age 22.0±2.0 years; range 19–29). Patients with organic diseases were excluded. In addition, 142 healthy volunteers (78 males and 64 females) were recruited at Tohoku University as controls (mean age 22.0±2.3 years; range 19–32). Subjects without any symptoms or signs with medical interview and physical examination were identified as healthy controls. IBS patients were diagnosed according to Rome III criteria [30]. In brief, IBS was defined as recurrent abdominal pain or discomfort at least 3 days per month in the last 3 months associated with two or more of the following symptoms: improvement with defecation, onset associated with a change in frequency of defecation, and/or onset associated with a change in form (appearance) of stools. These criteria were fulfilled for the previous 3 months with symptom onset at least 6 months prior to diagnosis. According to Rome III criteria, IBS was classified as IBS with diarrhea (D), constipation (C), or mixed symptoms of diarrhea and constipation (M). Unclassified IBS patients were classified as IBS-M. All subjects provided written informed consent and this study was approved by the Tohoku University Ethics Committee. Serial patients who agreed to participate in this study were enrolled.

### Evaluation of negative emotion

Emotional state was rated using the Perceived Stress Scale (PSS) [31], [32], the State–Trait Anxiety Inventory (STAI) [33], [34], and the Self-rating Depression Scale (SDS) [35], [36]. The Japanese versions of STAI, SDS, and PSS have been well validated and their reliability has been confirmed [32], [34], [36].

### Genotyping

**Figure 1:**
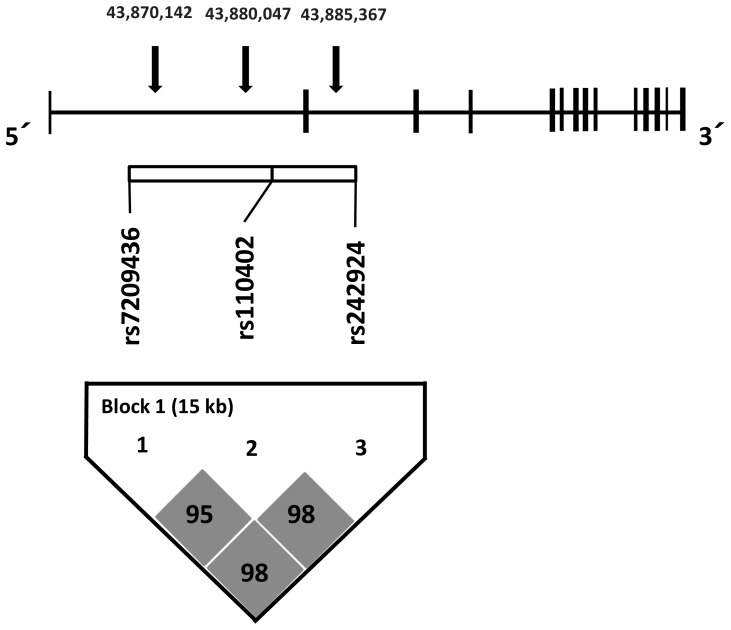
SNPs of the *CRH-R1* gene examined in this study. *CRH-R1* is located on chromosome 17q21.31. SNPs of *rs7209436, rs242924, and rs110402* are covering the gene in first and second intron on 5′ end including promoter region, a total region of 51.55 kb that has links with haplotype block. The structure was determined using the confidence interval method in Haploview such that we were able to estimate haplotypes for every participant with a posterior probability of >0.998. These SNPs had a minor allele frequency of >1.0% in the Japanese population.

Peripheral blood was collected from the forearm vein of each subject with a heparinized syringe. DNA was then extracted from the lymphocytes using a standard protocol [37]. Three SNPs (*rs110402*, *rs242924*, *rs7209436*) in the regulatory region of the *CRH-R1* gene were genotyped using direct sequencing and TaqMan real-time polymerase chain reaction (PCR) (**Figure 1**).

PCR amplification was carried out using the following primer pairs designed with primer3 version 4.0. (http://frodo.wi.mit.edu/primer3/): *rs110402*, 5′- AGA GCA AGA GGT GGC ACA G-3′ and 5′- CTA AGT GCT CTA CTT GTG AGC CTC-3′; *rs242924*, 5′-GAA ACT GAG GCA TGG GAG AG-3′ and 5′-CCA CAT CTC ATG GTA GCT GC-3′; *rs7209436*, 5′-CCT TTG TTC TCA CCT CAT CC-3′ and 5′-GGA TTT GTG ACT CAA CGG CT-3′. We performed PCR amplification using a total volume of 50 µl solution consisting of 0.2 µM of each primer, 1.25 U Prime STAR HS DNA Polymerase, 200 µM deoxynucleotide triphosphate, 1× Prime STAR buffer, and recombinant Taq DNA Polymerase (TAKARA BIO INC., Shiga, Japan). After initial denaturation at 94°C for 4 min, amplification was performed using 35 cycles at 94°C for 1 min (denaturation), 60°C for 1 min (annealing), and 72°C for 1 min (extension), followed by final elongation at 72°C for 7 min. Amplification products were separated on 2% agarose gel by electrophoresis. PCR products were purified from agarose gel using a QIA quick Gel Extraction Kit (Qiagen, Hilden, Germany). Amplimers were sequenced directly using the ABI PRISM dRodamine TM Terminator Cycle Sequencing Ready Reaction Kit (PE Applied Biosystems, Foster City, CA), and excess dye terminators were removed using CENTRI-SEP Columns (Princeton Separations, Adelphia, NJ).

Direct sequencing was performed on ABI 3130 Genetic Analyzer (PE Applied Biosystems). Subjects were also genotyped with amplification of the endpoint fluorescence using PCR with the CX-96TM Real-time PCR Detection System (Bio-Rad laboratories Inc., Tokyo, Japan). The primer set of C157008710 for *rs7209436* was purchased from Applied Biosystems. Primers for *rs110402* and *rs242924* were designed as follows: FAM, 5′-TTT CTT TGC ATA ACG CAA CAC CAG TCC TC-3′ and HEX, 5′-TTT CTT TGC ATA ACA CAA CAC CAG TCC TC-3′; and FAM, 5′-CTG GGC AAA AAT GGA GAG GGT CCC TG-3′ and HEX, 5′-CTG GGC AAA AAT GTA GAG GGT CC CTG-3′, respectively. After initial denaturation at 95°C for 3 min, amplification was performed using 49 cycles at 95°C for 10 s, 69.0–69.5°C for 30 s (annealing), and 95°C for 10 s. Individual alleles were measured using TaqMan Genotyping Master Mix (Applied Biosystems, Catalog 4371357) for *rs7209436* and SsoFast Probe Supermix (BioRad, Catalog 1725230) for *rs110402* and *rs242924*. All procedures were performed according to the manufacturer's instructions.

### Statistical analysis

We used Haploview [38] to determine the linkage disequilibrium (LD) structure of the SNPs within the *CRH-R1* gene and test for Hardy–Weinberg equilibrium. We also compared the LD structure of a subgroup of *CRH-R1* SNPs. The genotypes, alleles, and TAT haplocopies of *CRH-R1* SNPs were compared between IBS patients and controls, or between patients with different bowel patterns (normal, constipation, mixed, and diarrhea) using the chi-squared test. The effects of variation in *CRH-R1* SNPs and TAT haplocopies on emotional states were examined with two-way analysis of variance (ANOVA). A post hoc test was performed to determine the significance of genotype effects. Statistical analyses were performed using SPSS PASW Statistic version 18.0 software (IBM Inc., New York, NY). Results are expressed as mean ± S.E., and *P*<0.05 was considered significant.

## Results

Using Haploview we were able to estimate haplotypes for every participant with a posterior probability greater than 0.998, which allowed us to assign a score of 0, 1, or 2 copies of the TAT haplotype to every individual with a very high degree of certainty. The TAT haplotype accounted for 84% (410/245×2) of all haplotypes in the sample, with its complement CGG accounting for the remaining 16% (80/245×2).

**Figure 2:**
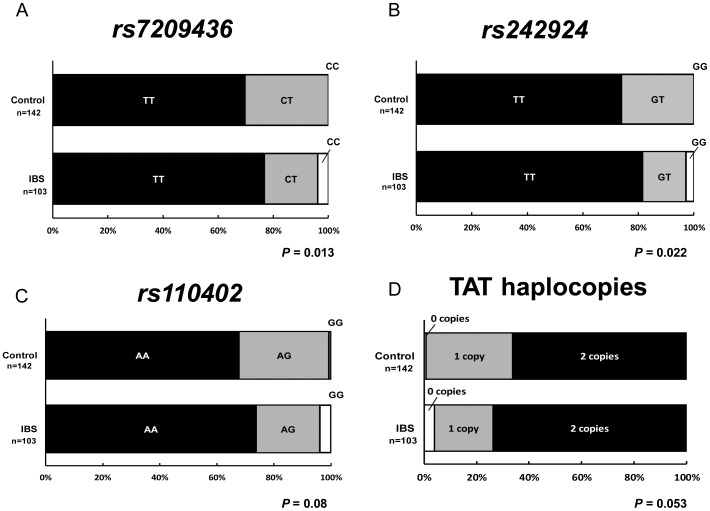
Difference in genotype of *CRH-R1* SNPs between controls and IBS patients. The SNPs *rs7029436* (**A**), *rs242924* (**B**), and *rs110402* (**C**), and TAT haplotype (**D**) were shown. The SNPs *rs7029436* (*P* = 0.013) and *rs242924* (*P* = 0.022, χ^2^-test) in IBS patients significantly differed from those in controls.

**Table 1** shows the genotype distribution and **Table 2** shows the allele frequency. Sex was not significantly associated with the SNPs and number of TAT haplotypes. The *rs7209436* TT genotype was significantly more common in IBS patients than in controls (χ^2^_(2)_ = 8.66, *P* = 0.01) (**Figure 2A**) and *rs242924* (χ^2^_(2)_ = 7.64, *P* = 0.02) (**Figure 2B**) but not on *rs110402* (**Figure 2C**). TAT haplotype copies were tendentially but not significantly different between IBS and controls (χ^2^_(2)_ = 5.88, *P* = 0.053) (**Figure 2D**).

**Table 1:** Genotypes and haplotype frequencies for three *CRHR1* SNPs in IBS patients and controls.

		Controls n (%) n = 142		IBS Patients n (%) n = 103		*P* value
		Male (n = 78)	Female (n = 64)	Male (n = 43)	Female (n = 60)	Control vs. IBS
**rs7209436**	**TT**	53 (37.3)	46 (32.4)	34 (33.0)	45 (43.7)	0.013
	**CT**	25 (17.6)	18 (12.7)	9 (8.7)	11 (10.7)	
	**CC**	0 (0.0)	0 (0.0)	0 (0.0)	4 (3.9)	
**rs110402**	**AA**	51 (35.9)	45 (31.7)	34 (33.0)	42 (40.8)	0.076
	**AG**	26 (18.3)	19 (13.4)	9 (8.7)	14 (13.6)	
	**GG**	1 (0.7)	0 (0.0)	0 (0.0)	4 (3.9)	
**rs242924**	**TT**	59 (41.5)	46 (32.4)	37 (35.9)	47 (45.7)	0.022
	**GT**	19 (13.4)	18 (12.7)	6 (5.8)	10 (9.7)	
	**GG**	0 (0.0)	0 (0.0)	0 (0.0)	3 (2.9)	
**TAT haplotype**	**0 copies**	1 (0.7)	0 (0.0)	0 (0.0)	4 (3.9)	0.053
	**1 copy**	26 (18.3)	21 (14.8)	9 (8.7)	14 (13.6)	
	**2 copies**	51 (35.9)	43 (30.3)	34 (33.0)	42 (40.8)	

**Table 2:** Allele expressions in controls and IBS patients and IBS subtypes for three SNPs of the *CRH-R1* gene.

		Controls	IBS patients				Total
			All	C	M	D	
		(n = 142)	(n = 103)	(n = 32)	(n = 31)	(n = 40)	(n = 245)
**rs7209436**	**T allele−**	0	4	2	2	0	4
	**T allele+**	142	99	30	29	40	241
	**C allele−**	99	79	22	26	31	178
	**C allele+**	43	24	10	5	9	67
**rs110402**	**A allele−**	1	4	2	2	0	5
	**A allele+**	141	99	30	29	40	240
	**G allele−**	96	76	21	25	30	172
	**G allele+**	46	27	11	6	10	73
**rs242924**	**T allele−**	0	3	2	1	0	3
	**T allele+**	142	100	30	30	40	242
	**G allele−**	105	84	23	27	34	189
	**G allele+**	37	19	9	4	6	56

IBS subtype: C, constipation; M, mixed; D, diarrhea.

**Figure 3:**
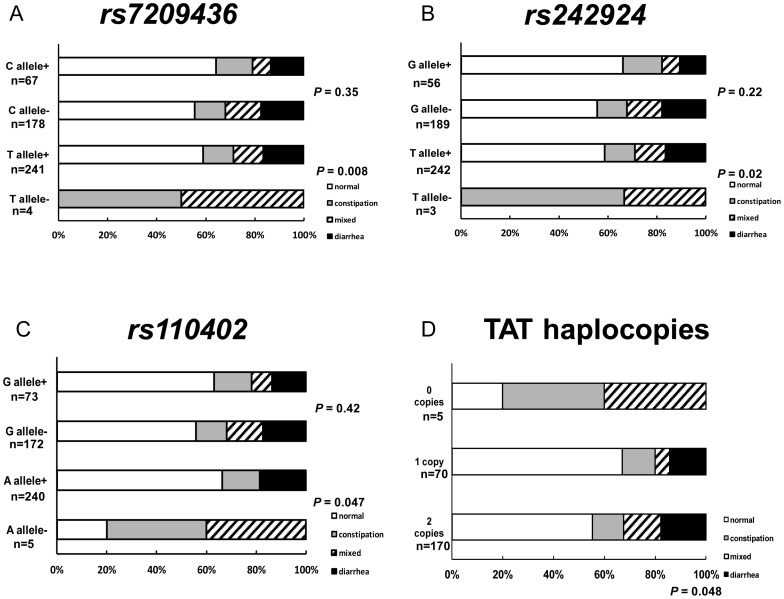
Difference in bowel patterns between allele of *CRH-R1* SNPs. Each panel indicates bowel patterns (normal, constipation, mixed, or diarrhea) in the SNPs of (**A**) *rs7029436* (C+ vs C−, T+ vs T−), (**B**) *rs242924* (G+ vs G−, T+ vs T−), and (**C**) *rs110402* (G+ vs G−, A+ vs A−) and (**D**) TAT haplocopies (0, 1, or 2 copies). Significant differences in bowel patterns between the T alleles of *rs7029436* (*P* = 0.008), the T alleles of *rs242924* (*P* = 0.02), the A alleles of *rs110402* (*P* = 0.047), and among TAT haplocopies (*P* = 0.048, χ^2^-test) of *CRH-R1* SNPs were observed.

There were no significant associations between IBS subtypes (D, C, and M) and genotypes of the three SNPs. However, bowel habit pattern (normal, D, C, and M) was significantly associated with T allele expression of *rs7209436* (χ^2^_(3)_ = 11.75, *P* = 0.008) (**Figure 3A**), T allele expression of *rs242924* (χ^2^_(3)_ = 9.97, *P* = 0.019) (**Figure 3B** **)**, A allele expression of *rs110402* (χ^2^_(3)_ = 7.96, *P* = 0.047), (**Figure 3C**), and TAT haplocopies (χ^2^_(6)_ = 12.68, *P* = 0.048) (**Figure 3D**).

**Table 3** shows that there are associations between TAT haplotype copy number and group (IBS patients vs. controls), gender (males vs. females), and bowel patterns (normal, D, C, and M). There was a significant gender effect in IBS patients with two copies of the TAT haplotype, as evidenced by more diarrhea in men and more constipation/mixed symptoms in women (χ^2^_(2)_ = 17.17, *P* = 0.001). Of the genotypes, the TT of *rs7209436* (*P* = 0.001, Fisher's test) and *rs242924* (*P* = 0.001, Fisher's test), and the AA allele of *rs110402* (*P* = 0.001, Fisher's test) showed a significant predominance of diarrhea in men and female dominance of constipation/mixed symptoms in women.

**Table 3:** IBS subtype (bowel pattern) in relation to TAT haplotype copy number and sex.

TAT copies		Control (n = 142)	IBS patients (n = 103)			Association with TAT haplotype copies *P* value		
			C (n = 32)	M (n = 31)	D (n = 40)	Control vs. IBS	Bowel patterns	Male vs. female
**0**	**Male**	1	0	0	0	0.05	0.048	0.082
	**Female**	0	2	2	0			
**1**	**Male**	26	3	2	4			0.58
	**Female**	21	6	2	6			
**2**	**Male**	51	4	8	22			0.001
	**Female**	43	17	17	8			

IBS subtype: C, constipation; M, mixed; D, diarrhea.

**Figure 4:**
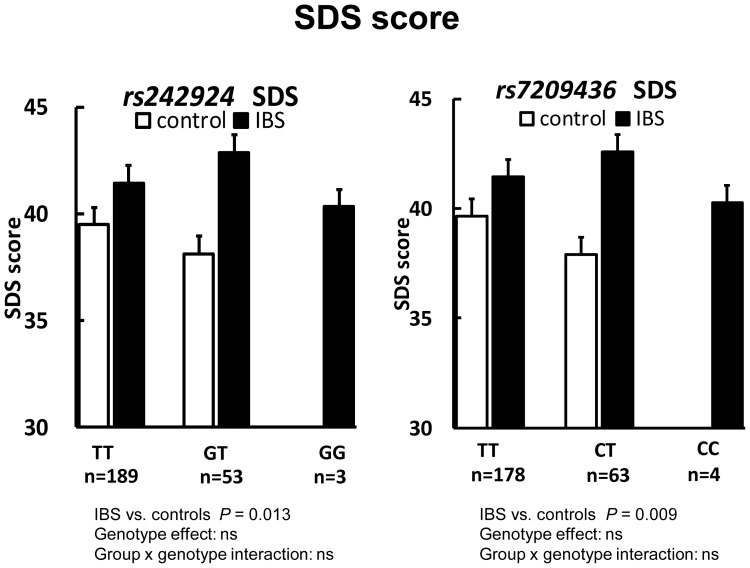
Self-rating Depression Scale and *CRH-R1* SNPs. Two-way ANOVA showed that IBS patients with *rs7029436* (*P* = 0.009) (right) and rs242924 (*P* = 0.013) (left) had significantly higher depression scale scores than controls with the same genotypes. However, there was no gene–group interaction.

The SDS score in IBS patients was significantly higher than in controls (*P* = 0.02) (**Table 4**). The PSS score in IBS patients was also significantly higher than in controls (*P* = 0.03). The STAI scores in IBS patients did not differ from those in controls. The two-way ANOVA indicated that the SDS scores in IBS patients were significantly higher than in controls despite the *rs242924* genotypes (TT, GT, and GG) (*P* = 0.013) and the *rs72094364* genotypes (TT, CT, and CC) (*P* = 0.009) (**Figure 4**). However, among both IBS patients and controls, there were no significantly different interactions between *rs7209436*, *rs110402*, and *rs242924* genotypes. Similarly, there were no significantly different allele distributions or psychological scores.

**Table 4:** Perceived stress, depression, and anxiety in controls and IBS patients.

	Controls (n = 142)		IBS patients (n = 103)		
	Mean	SD	Mean	SD	*P* value
**SDS**	39.1	8.5	41.6	4.2	0.02
**PSS**	26.3	9.2	28.8	8.2	0.03
**STAI (state)**	44.2	9.7	45.7	9.2	0.22
**STAI (trait)**	46.6	10.9	49.2	10.3	0.07

SDS: Self-rating Depression Scale, PSS: Perceived Stress Scale,

STAI: StateTrait Anxiety Inventory.

## Discussion

To our knowledge, the present study is the first to show an association between IBS symptoms and SNPs of the *CRH-R1* gene. Our finding of an increased frequency of the TT genotype in *rs7209436* and *rs242924* supports the main hypothesis that genetic polymorphisms and haplotypes of *CRH-R1* control the IBS phenotype. The genotype and allele frequency of *CRH-R1* SNPs in Japanese general population are presented in the database (Hap Map Project: http://hapmap.ncbi.nlm.nih.gov/index.html.en). TT genotype of *rs7209436* and *rs242924* are around 70% of normal population. Therefore, increased frequency of TT genotype of *rs7209436* and *rs242924* alone cannot explain relationship between *CRH-R1* SNPs and IBS. However, IBS individuals are characterized by more TT, less CT, and more CC of *rs7209436* and more TT, less GT, and more GG of *rs242924* than controls in our study. These findings suggest more homozygous preference on *rs7209436* and *rs242924* SNPs in parents of IBS individuals, resulting either 0 or 2 copies of TAT haplotype. It is of great interest to see whether these findings are replicated in another population or not. Morevover, decreased intermediate (heterozygous) genotypes of *CRH-R1* SNPs may relate to fundamentals of pathophysiology of allostatic load [1] in IBS patients. Allostatic load is either repeated stress overtime, lack of adaptation, prolonged response, or inadequate response [1]. In other words, pathological response to stress is not only toward one direction with exaggerated and overactive response but also toward another direction with impaired and hypoactive response. Stress responsiveness and genotyping in IBS patients are promising issue in the near future.

SNPs of *rs7209436, rs242924, and rs110402* are covering the gene in the first and second intron on 5′ end including promoter region of *CRH-R1* gene [20], [21]. They form a haplotype block [22], [23], [24]. Earlier studies indicated the influence of child abuse, the above SNPs [22], and haplotype copy numbers [22], [23], [24] on adult depression. CRH plays a major role in negative emotion formation through the 5-hydroxytryptamine (5-HT, serotonin)-2 receptor signaling pathway [39]. CRH-R1 activation leads to increased numbers and sensitivity of 5-HT2 receptors on the cell membrane of post-synaptic neurons [39]. We previously reported the effect of the 5-HT transporter gene-linked polymorphic region on colorectal distension-induced activation of the anterior cingulate cortex [40]. We showed that overt anxiety, which is recognized by lexical processing, was not different between the genotypes. Because in the present study we also found an association between *CRH-R1* genotypes and IBS symptoms, but not negative emotion measured by psychometric tests, *CRH-R1* genotypes may affect mainly physical (e.g., brain-gut) reactivity to stressors.

The association between *CRH-R1* genotypes and bowel movement is also in line with a possible link between *CRH-R1* genotypes and brain-gut reactivity to stressors. T alleles of *rs7209436* and *rs242924*, and the A allele of *rs110402* mediated diarrhea, while the lack of these alleles mediated constipation. Similarly, an increased number of TAT haplocopies were associated with an increased prevalence of diarrhea, while fewer copies were associated with constipation. There is a high degree of CRH immunoreactivity as well as an abundance of CRH-R1 receptors in the gut [41], especially in the myenteric plexus [42], [43]. Systematic excitation of myentric neurons occurs after the application of CRH via CRH-R1 receptors [42], [43]. Administration of CRH causes diarrhea in rats, which mimics stress-induced diarrhea in IBS patients [44].

Among IBS patients, men with two copies of the TAT haplotype had more diarrhea while women had more constipation/mixed symptoms. Individual allele analyses also supported this finding; the T alleles of *rs7209436* and *rs242924*, and the A allele of *rs110402* predisposed toward more diarrhea in men and more constipation/mixed symptoms in women. These findings are not surprising as an increased prevalence of diarrhea in men with IBS and more constipation/mixed symptoms among women has previously been reported [45]. Because 73.8% of individuals with IBS had two copies of the TAT haplotype, these findings may simply reflect the fact that two copies are present in the majority of IBS patients. However, sexual dimorphism in the CRH system was recently recognized: chronic variable mild stress induced more CRH mRNA in the paraventricular nucleus of male rats while the same stress decreased the level of CRH peptide in female rats [46]. In male rats subjected to perinatal stress, CRH-R1 mRNA expression was significantly greater in the central amygdala and basolateral amygdala [47]. In female rats subjected to stress during the perinatal period, CRH-R1 mRNA expression was greater than controls only in the medial amygdala [47]. By contrast, the effect of sex on CRH and CRH-R1 signaling in the myenteric plexus is largely unknown. The findings in this study of possible sexual dimorphism in the CRH system need further clarification.

Contrary to our hypothesis, no clear association between negative emotion and *CRH-R1* SNPs was found. Our data indicate that depression and perceived stress, but not anxiety, increased IBS in patients regardless of *CRH-R1* genotype. This is partially consistent with our previous report [13] and the results of others [45]. However, mean SDS score in IBS patients was below 49 and within normal range in this study. The findings of recent studies have provided behavioral and neuroendocrine evidence of stress vulnerability in GG homozygous individuals of SNP *rs110402* in the *CRH-R1* gene [48]. Among GG homozygotes, activation in the subgenual anterior cingulate cortex was greater in participants with major depressive disorder compared with controls [48]. However, only 0.7% of controls and 3.9% of IBS patients were GG homozygous for *rs110402* in this study. Therefore, the lack of association between negative emotion and *CRH-R1* gene may be explained by the small numbers of GG homozygotes.

This study has several limitations. First, the number of subjects is small. However, the study with even smaller subject populations (n = 99) have reported SNP-phenotype analyses [48]. Second, endophenotypes in our study were more global than biological endophenotypes. Although it is of great interest to identify the association between *CRH-R1* polymorphisms and the presence of IBS or bowel patterns in IBS patients, biological endophenotypes should be identified in future studies. Finally, the function of *CRH-R1* SNPs remains incompletely understood. The studied SNPs are located in introns and they do not influence sequence of the CRH-R1 protein directly. However, they may influence alternative mRNA splicing [49], [50] and expression of protein [51]. CRH-R1 expression and activity is regulated at the gene level by mRNA alternative splicing that results in a number of CRH-R1 variants [52]. This process can generate putative CRH-R1 receptor variants with distinct structural and signaling properties [53], [54]. Moreover, intron 1 of *CRH-R1* contains 3 highly conserved regions that may have regulatory functions (according to the UCSC Genome Browser database, http://genome.ucsc.edu/)[22]. Because functional intronic regulatory elements have been reported for several genes, these *CRH-R1* intronic regions could affect transcriptional modulation of gene function [22]. For instance, the GG genotype of *rs110402* in *CRH-R1* presumably causes increased expression of CRH-R1 [48]. More research is necessary to solve these limitations.

In conclusion, our findings support the hypothesis that genetic polymorphisms and haplotypes of *CRH-R1* mediate IBS and related bowel patterns. However, we could not find a clear association between *CRH-R1* genotypes and negative emotion. Further studies on IBS and the CRH system are therefore warranted.
